# Impact of Intestinal Peptides on the Enteric Nervous System: Novel Approaches to Control Glucose Metabolism and Food Intake

**DOI:** 10.3389/fendo.2018.00328

**Published:** 2018-06-22

**Authors:** Anne Abot, Patrice D. Cani, Claude Knauf

**Affiliations:** ^1^NeuroMicrobiota, European Associated Laboratory (EAL), INSERM, Université catholique de Louvain (UCL), Toulouse, France; ^2^INSERM U1220 Institut de Recherche en Santé Digestive (IRSD), CHU Purpan, Université Toulouse III Paul Sabatier, Paris, France; ^3^Metabolism and Nutrition Research Group, Louvain Drug Research Institute (LDRI), WELBIO (Walloon Excellence in Life Sciences and BIOtechnology), Université catholique de Louvain (UCL), Brussels, Belgium

**Keywords:** enteric nervous system, bioactive peptides, food intake, glucose metabolism, diabetes

## Abstract

The gut is one of the most important sources of bioactive peptides in the body. In addition to their direct actions in the brain and/or peripheral tissues, the intestinal peptides can also have an impact on enteric nervous neurons. By modifying the endogenousproduction of these peptides, one may expect modify the “local” physiology such as glucose absorption, but also could have a “global” action *via* the gut–brain axis. Due to the various origins of gut peptides (i.e., nutrients, intestinal wall, gut microbiota) and the heterogeneity of enteric neurons population, the potential physiological parameters control by the interaction between the two partners are multiple. In this review, we will exclusively focus on the role of enteric nervous system as a potential target of gut peptides to control glucose metabolism and food intake. Potential therapeutic strategies based on *per os* administration of gut peptides to treat type 2 diabetes will be described.

## Overview of the Enteric Nervous System (ENS)

### Structural and Functional Organization of ENS

The ENS, referred to as the “second brain,” is composed of more than 600 million of neurons and glial cells in human. ENS runs along the gastrointestinal (GI) tract and is organized in two main plexuses. The submucosal plexus (or Meissner’s plexus) lies in the submucosa of the intestinal wall and the myenteric plexus (or Auerbach’s plexus) between the longitudinal and circular layers of the external musculature. Nerve fiber bundles connect the ganglia within a plexus and between the different plexi (1). Despite some differences in structural organization along the GI tract, the ENS controls main regulator of GI functions such as secretion, barrier function, and movement of fluid across the lining epithelium. It is also a key regulator of local blood flow, interaction with the immune and endocrine systems of the gut and intestinal motility (1).

**Figure 1:**
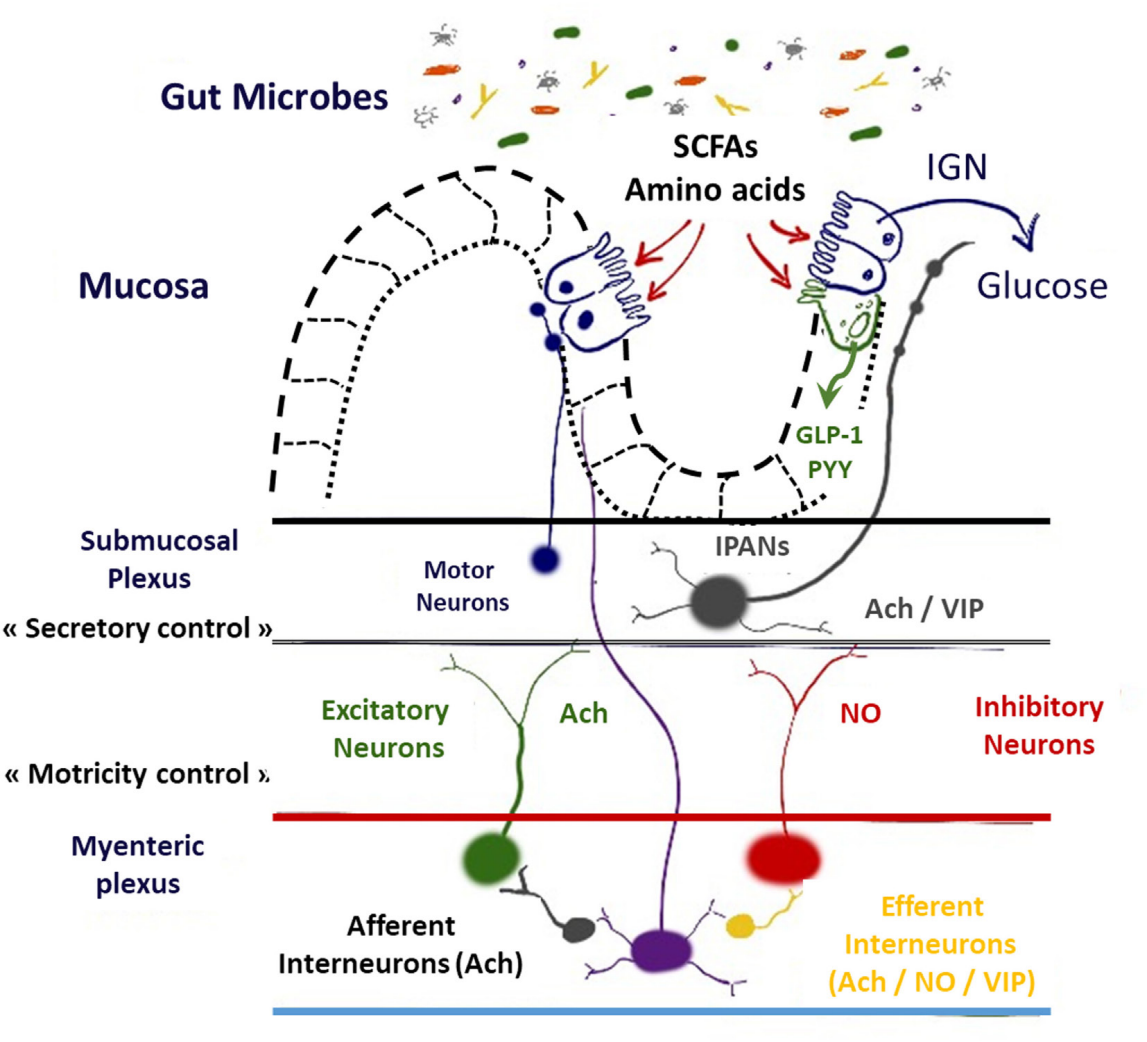
Schematic representation of interrelations between hormones, nutrients, neurotransmitters, and enteric nervous system. Abbreviations: SCFA, short chain fatty acids; GLP-1, glucagon-like peptide-1; PYY, peptide YY; IPAN, intrinsic primary afferent neurons; Ach, acetylcholine; NO, nitric oxide; VIP, vasoactive intestinal peptide; IGN, intestinal gluconeogenesis.

Motility reflexes, mainly regulated by myenteric neurons, are necessary for the physiology of digestion and to modulate gastric emptying and GI transit time (propulsion by peristaltic waves, induced by migrating motor complex, and mixing the chyme by segmentation, in fed conditions, along the digestive tract) and favor nutrients absorption (1, 2). After food intake, mechanical distortion and modification of luminal chemistry induced by the chyme activate first networks of interconnected intrinsic sensory neurons IPANs (for intrinsic primary afferent neurons) and MEN (for mechanosensitive enteric neurons) (3–5). Action potentials generated are conducted to synaptic connections with IPANs to interneurons and to excitatory/inhibitory motoneurons that innervate the muscle (6). Each functional type of enteric neurons is defined by a neurochemical code (1). The ENS is mostly composed of choline acetyl transferase and neuronal nitric oxide synthase (nNOS) neurons that, respectively, stimulate and inhibit intestinal smooth muscle cells [for review, see Ref. (7)] (Figure 1).

This system is closely connected to the central nervous system (CNS) *via* sensory neurons that send afferent fibers conveying afferent nervous message. After hypothalamic integration, efferent sympathetic and parasympathetic pathways can modulate ENS functions such as motility, secretion, and circulation (1).

### Impact of Nutrients on ENS Activity

Once nutrients have been absorbed or produced by metabolic activity, they are able to activate myenteric and/or submucosal neurons, encoding sensory stimuli, and therefore modulate ENS functions. In fact, enteric neurons express transporters and receptors which are involved in sensing of main nutrients such as SGLT-1 (Na^+^–d-glucose transporter) for glucose sensing, G-protein coupled receptor (GPR) 41 and 43 for short chain fatty acids (SCFAs) sensing, Pept2 (dipeptide transporter) and amino acid receptors activated by glutamate, glycine, or GABA for protein/peptide sensing [for review, see Ref. (8, 9)].

### Physiopathological Aspect

Alteration of nutrients and/or hormones sensing in the intestine are associated with aberrant hypothalamic responses that could lead to pathological states including T2D (10–12). For example, obese/diabetic mice and humans exhibit a decrease of nNOS expression in the digestive tract in correlation with an alteration of the ENS activity associated with intestinal hyper-contractility (12–16). In fact, physiopathological mechanism of diabetes-induced GI enteric neuropathy is complex and multifactorial. Autonomic neuropathy can affect both afferent and efferent connections between ENS and CNS (10, 12). High-fat diet ingestion induces damages and plastic changes in myenteric neurons that disrupt neural circuits, causing symptoms of dysmotility and correlates with neuropathy in the myenteric plexus of obese mice with symptoms of type 2 diabetes from duodenum to colon (15, 17). Indeed, high-fat diet ingestion during 12 weeks in mice induces duodenal hyper-contractility, accelerates colonic transit with enhanced neuronal cholinergic and serotoninergic excitation. This is a key feature of a later phase of obesity and is involved in altered ENS functions and abnormal duodenal absorption and colonic transit (12, 16, 18).

## Peptides Modulating ENS Neurons

It is well known that numerous bioactive peptides from different origins are able to modulate ENS activity. They are present in the intestinal lumen and can reach the myenteric plexus to modulate the activity of ENS neurons (12, 16, 19). Due to the great number of bioactive peptides able to control ENS (see Table 1), we decided to focus on some particular intestinal and bacterial peptides which present a potential therapeutic action on metabolic and feeding disorders.

**Table 1:** Hormones and neuropeptides implicated in the control of enteric nervous system (ENS).

Peptide nature	Peptide	Impact on ENS functions	Mechanism of action on enteric neurons	Experimental model	Reference
Hormones	Proglucagon-derived peptides	Inhibition of the spontaneous and evoked mechanical activity of duodenum and colon [glucagon-like peptide-1 (GLP-1)]	Decrease of the excitatory cholinergic neurotransmission through presynaptic GLP-IRs and modulation of NO release	*Ex vivo* conditions (mice)	Amato et al. (20)

		Reduction of gastric motility and gastric emptying (GLP-1)	Exogenous GLP-1 acts in the antral region, through neural NO release	*Ex vivo* conditions (mice)	Rotondo et al. (21)

		Reduction of gastric motility and gastric emptying (GLP-2)	Exogenous GLP-2 increases locally GLP-2R expression depending on fed conditions	*In vivo* conditions (normal and diabetic mice)	Rotondo et al. (21)

		Neuroprotective effects (GLP-1 and GLP-2)	Improvement of myenteric neurons survival in apoptose-induced conditions	*In vitro* conditions (cultured myenteric neurons from rat small intestine)	Voss et al. (22)

		Reduction intestinal mucosal inflammation (GLP-2)	Activation of vasoactive intestinal polypeptide (VIP) neurons of the submucosal plexus, reduced levels of inflammatory cytokines (IFN- gamma. TNF-alpha. IL-lbeta) and inducible nitric oxide (NO) synthase, with increased levels of IL-10	*In vivo* conditions (colitis rat model)	Sigalet et al. (23, 24)

		Luminal infusion of cholecystokinin (CCK) can produce segmenting activity in duodenum and jejunum	Involvement of CCK-1 and CCK-2 receptors and serotoninergic pathway in the mucosa	*Ex vivo* conditions (guinea pig small intestine)	Ellis et al. (25)

	CCK	Promotion of oxytocin-induced contractions of longitudinal muscle ships of duodenum	Exogenous oxytocin favor the duodenal CCK release from the neurons of the myenteric plexus to inhibit the muscle contraction	*Ex vivo* conditions (rats)	Lv et al. (26)

	Ghrelin	Peripheral administration of exogenous ghrelin enhance fasted motor activity of the gastrointestinal tract	Stimulation of enteric cholinergic neurons + possible role of serotonin	*In vivo* conditions (human: antra duodenal manometry/record in freely moving conscious rats) and *ex vivo* conditions (for mechanism, in mice)	Human: Tract et al. (27)/rat: Fujino et al. (28) and Taniguchi et al. (29)/mouse: Edholm et al. (30) and Yang et al. (31)

	Leptin	Modulation of the activity of enteric inhibitory and excitatory neurons in proximal colon.	Impact on enteric nitrergic neurons and intrinsic primary afferent neuron	*Ex vivo* conditions (rats)	Florian et al. (32)

	Apelin	Inhibition of duodenal contractions	Stimulation of the activity of duodenal neuronal NO synthase neurons	*Ex vivo* conditions (diabetic mice)	Foumel et al. (12)

Neuropeptides	Peptide YY (PYY)	Stimulation of propulsive colonic motor function	PYY inhibits basal and serotoninergic and cholinergic on myenteric neurons of the descending colon	*In vivo* conditions (microtransducer in conscious mice model)	Browning et al. (33) and Wang et al. (34)

		Stimulation gastric motor activity	Activation of enteric excitatory neurons releasing acetylcholine and tachykinins	*In vivo* conditions (mice)	Amato et al. (35)

		Stimulation of both enteric plexus	PYY injected intraperitoneally activates small intestinal enteric neurons, both myenteric and submucosal	*In vivo* conditions (rat)	Newman et al. (36)

	Neuropeptide Y (NPY)	Regulation of the inflammation NPY severity and indirectly gut motility	Upregulation of NPY in ENS and neuropeptide Y1 receptor	*In vivo* conditions (colitis mouse model)	Wheway et al. (37)/Chandrasekharan et al. (38, 39)

	Galanin	Improvement of myenteric neurons survival in apoptose-induced conditions Inhibition of duodenal contractions	Activation of nitrergic myenteric neurons Improvement of NO release of enteric neurons	*In vitro* (cultured myenteric neurons from rat small intestine) *Ex vivo* conditions (diabetic mice)	Arciszewski et al. (40) Abot et al. (16)

### Intestinal Bioactive Peptides

#### Peptide Hormone

Among the specialized cell types localized to the intestinal epithelium, enteroendocrine cells (EECs) take part of the gut homeostasis and the efficiency of nutrient absorption through secretion of specialized peptide hormones that act in an autocrine, paracrine, or endocrine manner and inform to nutrient availability (41). Different specialized EEC subtypes localized along the GI tract expressing distinct peptide families have been identified (42).

##### Proglucagon-Derived Peptides (PGDPs)

The PGDPs, notably glucagon-like peptide-1 (GLP-1) and GLP-2 are secreted in response to meal ingestion predominantly by EEC L-cells located along the GI tract (43, 44). This gut PGDPs exhibit robust actions controlling gut motility, but also food intake and glucose homeostasis by reducing postprandial glycemia (45, 46). GLP-1 exerts not only inhibitory effects on GI motility and participates to glucose absorption through vagal afferents and central nervous mechanisms but also a direct influence on the GI wall from duodenum to colon (20, 45, 47) (Table 1). In fact, GLP-1 exerts an inhibitory effect on the spontaneous and evoked mechanical activity in the duodenum and colon of mice by acting in the enteric neurons, to decrease the excitatory cholinergic neurotransmission through presynaptic GLP-1Rs, which modulate NO release (20). Through this mechanism, GLP-1 is able to reduce gastric motility (21). GLP-2 treatment improves survival of myenteric neurons from adult rat small intestine through anti-inflammatory actions and increase population of vasoactive intestinal peptide (VIP)-expressing enteric neurons (22, 23, 48). Thus, both GLP-1 and GLP-2 have neuroprotective effects (22).

##### Cholecystokinin (CCK)

Cholecystokinin is secreted by I-cells, principally localized in duodenum and proximal jejunum as well as enteric and CNS in response to feeding. CCK receptors (CCK-1 and CCK-2) are expressed in enteric neurons and involved in regulating nutrient-induced segmentation, with the participation of neuronal serotonergic signaling, activating both intrinsic and extrinsic primary afferent neurons to, respectively, initiate peristaltic and secretory reflexes and to transmit information to the CNS (25). Thus, CCK is co-expressed in the neurons of the myenteric plexus in duodenum with oxytocin and participates to the inhibition of spontaneous contraction of the muscle strips, and this effect is abolished in response to lorglumide, a CCK1 receptor antagonist (26) (Table 1). This hormone is already under investigation in view of developing therapies for the treatment of obesity and type 2 diabetes (49).

##### Ghrelin

Ghrelin is produced in gastric endocrine cells and exerts its effects by interacting with ghrelin receptors (growth hormone secretagogue receptor 1a or GHSR1a) expressed within the GI enteric plexus (30, 50). Previous studies have demonstrated that ghrelin (endogenous or peripheral administration) promotes gastric and small intestinal motility, particularly fasted motor activity in experimental animal models but also in human (27–29). In fact, ghrelin stimulates motility in the small intestine of rats through intrinsic cholinergic neurons (30, 31) (Table 1).

##### Apelin

Apelin is a bioactive peptide implicated in the control of glucose metabolism by improving insulin sensitivity in normal and diabetic mice (51). Apelin is also secreted by enterocytes and is released in the luminal part of gut to favor glucose absorption (52).

#### Neuropeptides

##### Pancreatic Polypeptides (PP) and Related Peptides

Neuropeptide Y (NPY) and peptide YY (PYY) are structurally related peptides that are considered to mediate inhibitory actions on GI motility and secretion. In the ENS, double immunofluorescence demonstrates that subpopulations of the Y1 receptor-positive nerve cell bodies are immunopositive for NPY, VIP, and NOS (53). Intraperitoneal injection of PYY and NPY inhibit fecal pellet output per hour and inhibited high-amplitude distal colonic contractions and cholinergic-stimulated propulsive colonic motor function (34). They can increase cFOS activity in enteric neurons and exert powerful inhibitory effects on myenteric neurons of the descending colon (33, 36). In proximal part of the GI tract, exogenous PP stimulates mouse gastric motor activity, by activating gastric enteric excitatory neurons releasing acetylcholine (Ach) and tachykinins (35) (Table 1).

##### Galanin

Galanin is a neuropeptide largely expressed in the brain but also in ENS neurons (54). In the intestine, the majority of galanin effects are mediated by Gal-R1 predominantly expressed in ENS neurons and more particular in neurons that express Ach, nNOS, or VIP. More precisely, galanin is known to be a neuropeptide which exerts an inhibitory action on myenteric cholinergic neurons and enhance nitrergic neurons activity (16, 55). Galanin also exerts positive effect on survival of cultured porcine myenteric neurons (40) (Table 1).

### Bacterial Bioactive Peptides

Microorganisms are able to synthesize a large number of metabolites which have beneficial or detrimental properties for human health. Interaction between gut microbiota and ENS in the control of gut motility and/or gut–brain axis is well documented. In fact, the gut microbiota produces bioactive molecules that act on enteric neurons to influence GI motility, and to modify the “gut–brain axis” by impacting on IPAN (56, 57). However, the exact biochemical nature of bacterial molecules implicated in the ENS could be extremely large. Among these, nitrogen bearing molecules from gut microbiota such as amino acids, amino acids derivatives, and oligopeptides have received great attention. Gut microbiota influences the biosynthesis and the release of enteric neurotransmitter such as serotonin, a monoamine that participates to the control of GI motility (58). Nowadays, little is known concerning the implication of bacterial bioactive peptides in the control of metabolism *via* ENS. In fact, signal peptides from bacteria are formylated, and some studies have demonstrated that *N*-formyl peptides from bacterial origin can be sense by host cells (59). *N*-formyl peptides are detected by formyl peptide receptors which are G-protein-coupled receptors that act as chemosensing receptors. No direct link between *N*-formyl peptides and ENS is described in the literature, but Cianciulli et al. have demonstrated that *N*-formyl-methionyl-leucyl-phenylalanine, synthetized by *Escherichia coli* and other Gram-negative bacteria, could release NO from chick embryo nerve cells (60).

## Peptides/ENS and Glucose Metabolism

As explain earlier, numerous peptides are able to modulate the activity of ENS neurons to control the contraction of intestinal smooth muscle cells. How the control of intestinal contraction could have an impact on glucose metabolism? First, the fed state is characterized by the presence of segmental waves in the proximal part of the intestine which favor glucose absorption. In fact, a positive correlation exists between intestinal contraction and glucose absorption (2). Second, the duodenal contraction can be detected by the hypothalamus *via* an afferent nervous signal (12). Consequently, the increase of duodenal contraction provokes a drastic decrease of hypothalamic NO release, and then decrease glucose entry in tissue (16). This information suggests that intestinal peptide, such as apelin (12), could modulate the “ENS-smooth muscle” couple to favor glucose absorption at the beginning of food intake (12). At the opposite, a high level of apelin can decrease duodenal contraction to block glucose absorption, and then to increase the release of hypothalamic NO to favor glucose entry in muscle (12). Here, this last result supposes that intestinal peptides could have an opposite effect at the end of food intake and/or digestion to limit the level of plasma glucose in the whole body.

During metabolic disorders, obese and diabetic patients and mice present an alteration of ENS neurons which has repercussions on colonic and duodenal contraction. Here, the duodenal hyper-contractility is associated with a significant increase of glucose absorption, and with a dysfunction of the gut–brain axis which favor an insulin resistant state (12). Nowadays, therapeutic strategies have just started to focus their attention on the impact of oral bioactive peptides to treat hyperglycemia and insulin resistance in diabetic patients (61). Recently, we have discovered that the chronic oral gavage of apelin in diabetic mice improves glucose tolerance and insulin sensitivity in skeletal muscle (12). At this high dose, apelin can stimulate the activity of duodenal nNOS neurons (1) first, to decrease glucose absorption by a “local” action and (2) second, to restore the gut–brain axis and then insulin sensitivity (12). Of course, apelin is now considered as an interesting potential candidate to treat type 2 diabetes *via* this novel mode of injection i.e., *per os* and/or by intravenous injection (61).

As opposed to apelin which could have a potential negative effect in the brain (62), another gut peptide candidate is well described in the literature. Galanin is a gut peptide that is also known as a neurotransmitter in the hypothalamus. Intracerebroventricular and i.v. injection of galanin in mice improve glucose homeostasis, and in particular insulin sensitivity (63–65). In the gut, the level of galanin is significantly decreased in the duodenum during type 2 diabetes (66). Recently, Abot et al. have discovered that chronic oral galanin treatment in diabetic mice have significantly improved glucose tolerance (16). Here, oral galanin is able to increase the release of enteric NO in the duodenum to restore the gut–brain axis similar to that previously observed with apelin. Finally, the improvement of glucose metabolism is associated with a significant increase of glucose entry in muscle, liver and adipose tissue.

How could the gut peptide reach the ENS and how can we use them as potential anti-diabetic drugs? Whether or not other mode of transport could exist in the intestine for these peptides is not characterized. The mechanisms used by luminal peptide to join the ENS neurons are not well described. Like that observed with leptin (19), apelin could reach the ENS *via* transcytosis. In a pharmacological point of view, peptides given orally must be active only in the duodenum to limit potential “indesirable” effect. For example, oral peptides could have a significant impact on gastric emptying and these could have consequences on digestion and/or food intake.

## Peptides/ENS and Food Intake

As discussed earlier in this review, the regulation of food intake is a complex and tightly regulated system requiring the integration of local (i.e., intestinal, ENS) signals and distal signals (i.e., in the brain). As resume recently by Prinz and Stengel (67), numerous gut peptides are able to control food intake. These peptides may exert anorexigenic (GLP-1, PPY, and CCK) or orexigenic (Ghrelin) effects by acting directly in the brain or by using an afferent vagal pathway. As these modes of action of gut peptides are well described in the literature and potentially do not require the ENS, we have decided to focus our attention on more recent concepts showing interactions between gut peptides, intestinal actors (epithelial and endocrine cells, enteric neurons, and microbiota), and food intake.

The role of the endogenous production of glucose by the intestinal epithelial cells may constitute a major mechanism by which nutrients regulate food intake. Several studies led by Pr. Mithieux and his team have elegantly demonstrated that the so called, intestinal gluconeogenesis (IGN), is a key process promoting a decrease of hunger but also an improvement of insulin sensitivity while the liver is decreasing is endogenous glucose production (68). IGN is a process that has been shown to be activated by the degradation of dietary proteins in amino acids (69). Interestingly, the same team has found that mice deficient of the key enzyme involved in the IGN (i.e., glucose-6 phosphatase) exhibit increased basal sympathetic tone but also develop hypothalamic resistance to leptin, suggesting again a key link with the regulation of food intake and energy homeostasis.

Remarkably, IGN is massively induced after metabolic surgery and can finally account for up to 20% of the total endogenous glucose production in post-absorptive state (70). It is worth noting that metabolic surgery (e.g., RYGB) is associated with a dramatic change in the route of the food nutrients and therefore may directly affect the gut microbiota composition (71). Gastric bypass is also characterized by a massive increase in gut peptides involved in food intake and glucose metabolism such as GLP-1 and PYY (72). This phenomenon can probably be attributed in part to the direct contact between nutrients (i.e., glucose, lipids, and amino acids) and EECs producing those hormones but also *via* the fermentation of non-digested food components and nutrients by the gut microbiota. One of the key examples linking the fermentation of fibers and the secretion of GLP-1 has been published many years before. Indeed, it has been clearly demonstrated that the mechanisms by which specific dietary fibers such as prebiotics (i.e., inulin, oligofructose) reduces glycemia, food intake and improves insulin sensitivity directly rely on the production of the gut peptide GLP-1 (73, 74). More recently, it has been shown that the metabolites produced by gut bacteria, such as the SCFAs butyrate and propionate, likely contribute to the modulation of the production of both GLP-1 and PYY in these conditions (75) (Figure 1).

It is important to note that all the aforementioned mechanisms, such as the IGN, the bacterial fermentation (i.e., SCFAs), or the gut peptides production, are connected to the ENS. For example, in the case of the fermentation of fibers, the generated compound propionate has been shown to bind to GPR-41 expressed in the nerve endings of the portal vein wall. But it has also been discovered that propionate production by microbes also controls IGN and eventually food intake (76) (Figure 1).

Finally, this last example clearly shows that all these interesting mechanisms are intertwined. Indeed, a recent study shows that VIP induces glucose-6 phosphatase activity and eventually IGN *in vivo*. VIP neurons innervate the gut epithelium, in addition, in this study the authors suggest that VIP is also important for the bacterial metabolite propionate-induced activation of IGN. Thus, because VIP is expressed in neurons present in submucosal and myenteric plexuses, and is modulated by the microbiota derived compounds produced by the fermentation. These findings also suggest that it exist a local release of VIP by enteric neurons which may be a key mechanism by which nutrients, peptides, and microbial metabolites are finally involved in the activation of IGN and thus control food intake and glucose metabolism (77).

## Summary/Conclusion

Intestinal bioactive peptides by acting on ENS neurons represent a potential therapeutic target to treat metabolic and food disorders associated with type 2 diabetes and obesity. Their origins, i.e., bacterial and/or intestinal wall facilitates their utilization for oral treatment. Whether their use alone or in combination with pre- and probiotics for therapeutic strategies will be tested in the future. Thus, the study of the gut–brain axis is only at the beginning of its story, and bioactive peptides represent one, but not the least, of the way forward to treat multiple diseases.

## Author Contributions

All authors listed have made a substantial, direct, and intellectual contribution to the work and approved it for publication.

## Conflict of Interest Statement

The authors declare that the research was conducted in the absence of any commercial or financial relationships that could be construed as a potential conflict of interest.
